# The use of venous Doppler to predict adverse kidney events in a general ICU cohort

**DOI:** 10.1186/s13054-020-03330-6

**Published:** 2020-10-19

**Authors:** Rory Spiegel, William Teeter, Scott Sullivan, Keegan Tupchong, Nabeel Mohammed, Mark Sutherland, Evan Leibner, Philippe Rola, Samuel M. Galvagno, Sarah B. Murthi

**Affiliations:** 1 Department of Critical Care, Georgetown University Medstar Washington Hospital Center, Washington, DC USA; 2 Department of Emergency Medicine, Georgetown University Medstar Washington Hospital Center, Washington, DC USA; 3 Department of Emergency Medicine, R Adams Cowley Shock Trauma Center, University of Maryland School of Medicine, Baltimore, MD USA; 4 The U.S. Army Command and General Staff College, Leavenworth, KS USA; 5 Division of Critical Care, Department of Emergency Medicine, University of Texas Health Science Center at Houston (UTHealth), Houston, TX USA; 6 Division of Critical Care, Department of Medicine, University of Texas Health Science Center at Houston (UTHealth), Houston, TX USA; 7 Department of Surgical Critical Care, Medstar Washington Hospital Center, Washington, DC USA; 8 Department of Critical Care and Emergency Medicine, University of Maryland Medical Center, Baltimore, MD USA; 9 Critical Care Medicine, Department of Emergency Medicine Mount, Sinai Hospital, New York, NY USA; 10 Intensive Care Unit, Santa Cabrini Hospital, Montreal, Canada; 11 Department of Anesthesiology, Program in Trauma, R Adams Cowley Shock Trauma Center, University of Maryland School of Medicine, Baltimore, MD USA; 12 Program in Trauma and Surgical Critical Care, R Adams Cowley Shock Trauma Center, University of Maryland School of Medicine, Baltimore, MD USA

**Keywords:** Ultrasound, Venous return, Venous congestion

## Abstract

**Background:**

Changes in Doppler flow patterns of hepatic veins (HV), portal vein (PV) and intra-renal veins (RV) reflect right atrial pressure and venous congestion; the feasibility of obtaining these assessments and the clinical relevance of the findings is unknown in a general ICU population. This study compares the morphology of HV, PV and RV waveform abnormalities in prediction of major adverse kidney events at 30 days (MAKE30) in critically ill patients.

**Study design and methods:**

We conducted a prospective observational study enrolling adult patients within 24 h of admission to the ICU. Patients underwent an ultrasound evaluation of the HV, PV and RV. We compared the rate of MAKE-30 events in patients with and without venous flow abnormalities in the hepatic, portal and intra-renal veins. The HV was considered abnormal if S to D wave reversal was present. The PV was considered abnormal if the portal pulsatility index (PPI) was greater than 30%. We also examined PPI as a continuous variable to assess whether small changes in portal vein flow was a clinically important marker of venous congestion.

**Results:**

From January 2019 to June 2019, we enrolled 114 patients. HV abnormalities demonstrate an odds ratio of 4.0 (95% CI 1.4–11.2). PV as a dichotomous outcome is associated with an increased odds ratio of MAKE-30 but fails to reach statistical significance (OR 2.3 95% CI 0.87–5.96), but when examined as a continuous variable it demonstrates an odds ratio of 1.03 (95% CI 1.00–1.06). RV Doppler flow abnormalities are not associated with an increase in the rate of MAKE-30

**Interpretation:**

Obtaining hepatic, portal and renal venous Doppler assessments in critically ill ICU patients are feasible. Abnormalities in hepatic and portal venous Doppler are associated with an increase in MAKE-30. Further research is needed to determine if venous Doppler assessments can be useful measures in assessing right-sided venous congestion in critically ill patients.

## Introduction

Fluid boluses are used to increase cardiac output (CO) and blood pressure in patients admitted to the intensive care unit (ICU) following surgery, trauma or septic shock [1]. There is a growing body of literature suggesting excess fluid administration is detrimental, leading to increased rates of acute kidney injury (AKI), prolonged days of mechanical ventilation and death [2]. One of the physiological underpinnings of this may be an increase in both right and left atrial pressures, resulting in venous congestion and tissue edema. To date, there are no clearly established ultrasound measures of venous congestion reflecting right-sided pressures and venous congestion which could be used to indicate when fluid administration is becoming harmful.

The Surviving Sepsis Campaign (SSC) guidelines suggest using assessments of volume responsiveness (VR), defined as an increase in cardiac output (CO) by 15%, as a guide to fluid administration [3]. VR-based strategies, while broadly advocated, have not been shown to improve outcome in ICU patients. Additionally, these strategies may promote over-resuscitation as most recommend continuing fluid administration until the patients are no longer VR, thereby reaching the asymptotic portion of the Frank–Starling curve indicating further fluid is less impactful. VR-based strategies do not assess elevations in right atrial pressure (RAP) or assess for venous congestion which could occur earlier. While elevations of left atrial pressure can be seen clinically with hypoxia, cephalization on chest X-ray and B-lines on ultrasound resulting from pulmonary edema, elevation in right-sided pressure is much more difficult to detect. The likely importance of right-sided pressures is supported by a number of recent studies suggesting that central venous pressure (CVP), a marker of venous congestion, more accurately predicts AKI than markers of perfusion, CO or oxygen delivery [4, 5]. Elevations in right-sided pressure cause changes in the Doppler venous flow patterns which also predict outcome. Beaubien-Souligny et al. found that dilation of the Inferior Vena Cava (IVC) alone was not predictive of renal dysfunction. It was only in patients with a dilated IVC and Doppler findings of venous return flow abnormality or venous congestion that the risk of renal dysfunction was identified (9). It is possible that right-sided venous flow changes detect clinically important elevations in right atrial pressure that lead to venous congestion and end organ injury.

Doppler flow patterns of hepatic veins (HV), portal vein (PV) and intra-renal veins (RV) are noninvasive and accurately identify early stages of right-sided venous congestion in patients who have cardiac dysfunction or have undergone heart surgery. Abnormalities in venous flow patterns after open heart surgery predict AKI, right heart strain and other post-surgical complications [6–9]. Similarly, abnormal flow patterns are consistently measured in congestive heart failure with elevated right atrial pressures [10]. If HV, PV and RV can be validated as reliable measures of elevated RAP, such indicators might have utility in modulating fluid resuscitation in other critically ill patient populations.

To date, no studies have examined the utility of HV, PV and RV in a critically ill cohort of patients admitted to the ICU or assessed the feasibility of including them in a point-of-care ultrasound (POCUS) exam. The goals of this study are to demonstrate the feasibility of HV, PV and RV measurement and determine the relationship between flow pattern changes and MAKE-30 events in a general ICU population.

## Methods

### Patient selection

The study was approved by the University of Maryland, Baltimore Institutional Review Board (IRB) according to international and national laws (approval number HP-00082080). We conducted a prospective observational study in adult patients generated from a convenience sample of patients admitted to our medical, surgical, trauma or neurotrauma ICUs. Patients were screened for eligibility between 8 and 4 pm on Monday–Friday. Patients were considered eligible if they could be scanned within 24-h of ICU admission. Those with end-stage renal disease on outpatient hemodialysis, a transplanted kidney or liver or those who were transitioning to comfort care were excluded. After consent was obtained, patients underwent a focused rapid echocardiographic evaluation (FREE) [11] which includes HV, PV and RV assessments.

### Sonography

All exams were performed by one of four critical care fellows with POCUS and transthoracic echocardiography training. Participating fellows underwent a 1-month clinical rotation in which they were trained in the FREE under the guidance of a Registered Diagnostic Cardiac Sonographer (RDCS) and a RDCS critical care attending. All Doppler findings were obtained during the end-expiratory phase of the patient’s respiratory cycle and with concurrent multi-lead ECG tracings.

### Blinding of data and outcomes

Given the observational design, the images, interpretations or recommendations for care were not discussed with or made available to the treating clinicians. Pre-determined clinical data were abstracted from the electronic medical record immediately prior to the performance of the study by the fellow performing the study (described below).

In order to address potential bias, Doppler waveforms were reviewed by two members of the research team (RS and SM) blinded to clinical outcomes. Patients in whom US images were missing or of insufficient quality to determine Doppler waveforms were excluded from the analysis concerning that specific waveform pattern.

### Ultrasound image acquisition and interpretation of waveform morphology

#### Hepatic Vein Doppler (HVD)

**Fig. 1:**
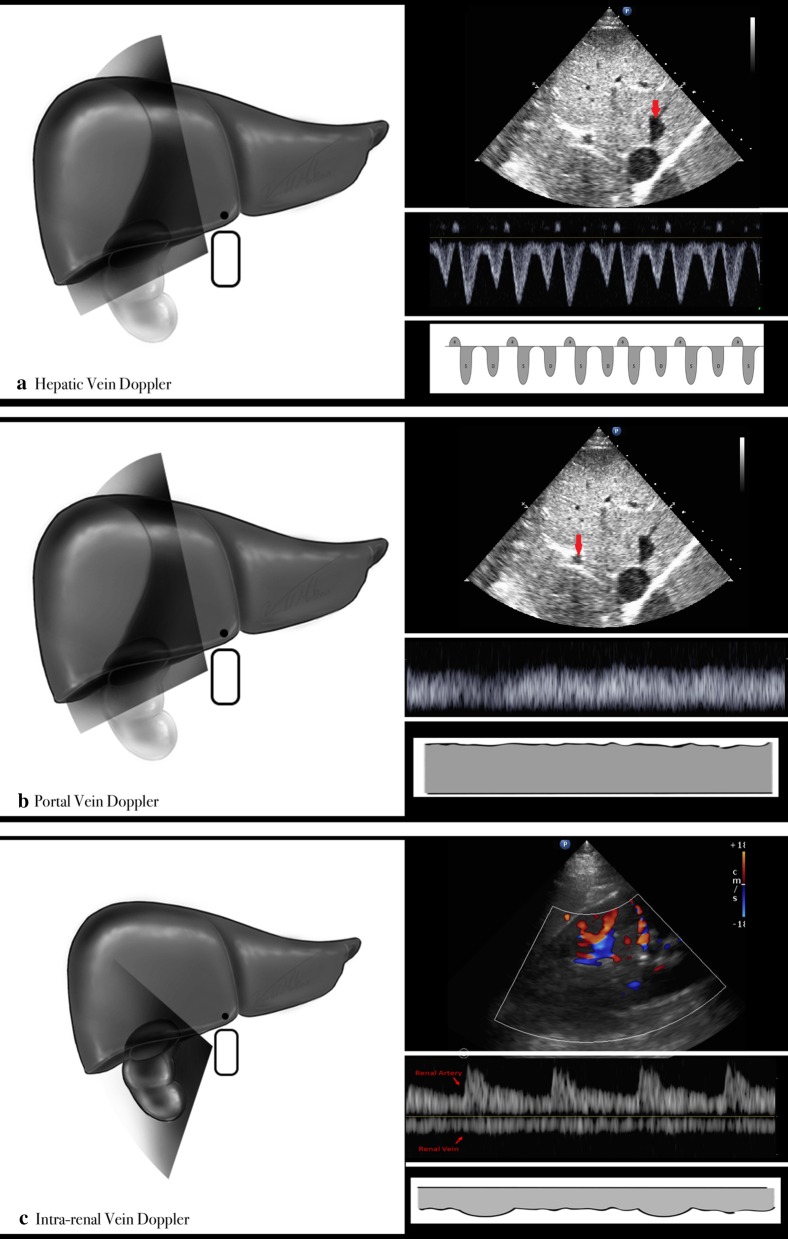
Ultrasound surface and B-mode anatomy and normal venous Doppler waveforms

To obtain the HV PW, a phased array transducer is used with cardiac pre-sets. ECG leads are placed to assist in the interpretation of HVD in sinus rhythm as well as atrial fibrillation. The middle hepatic vein is identified from a mid-subcostal or lateral views (Fig. 1a). It is interrogated 2–4 cm from its junction to the IVC. Color flow Doppler (CFD) is used to identify high flow parallel to the transducer, and the PW is obtained. The waveform is recorded, and the scale optimized. All Doppler findings were obtained during the end-expiratory phase of the patients respiratory cycle.

**Fig. 2:**
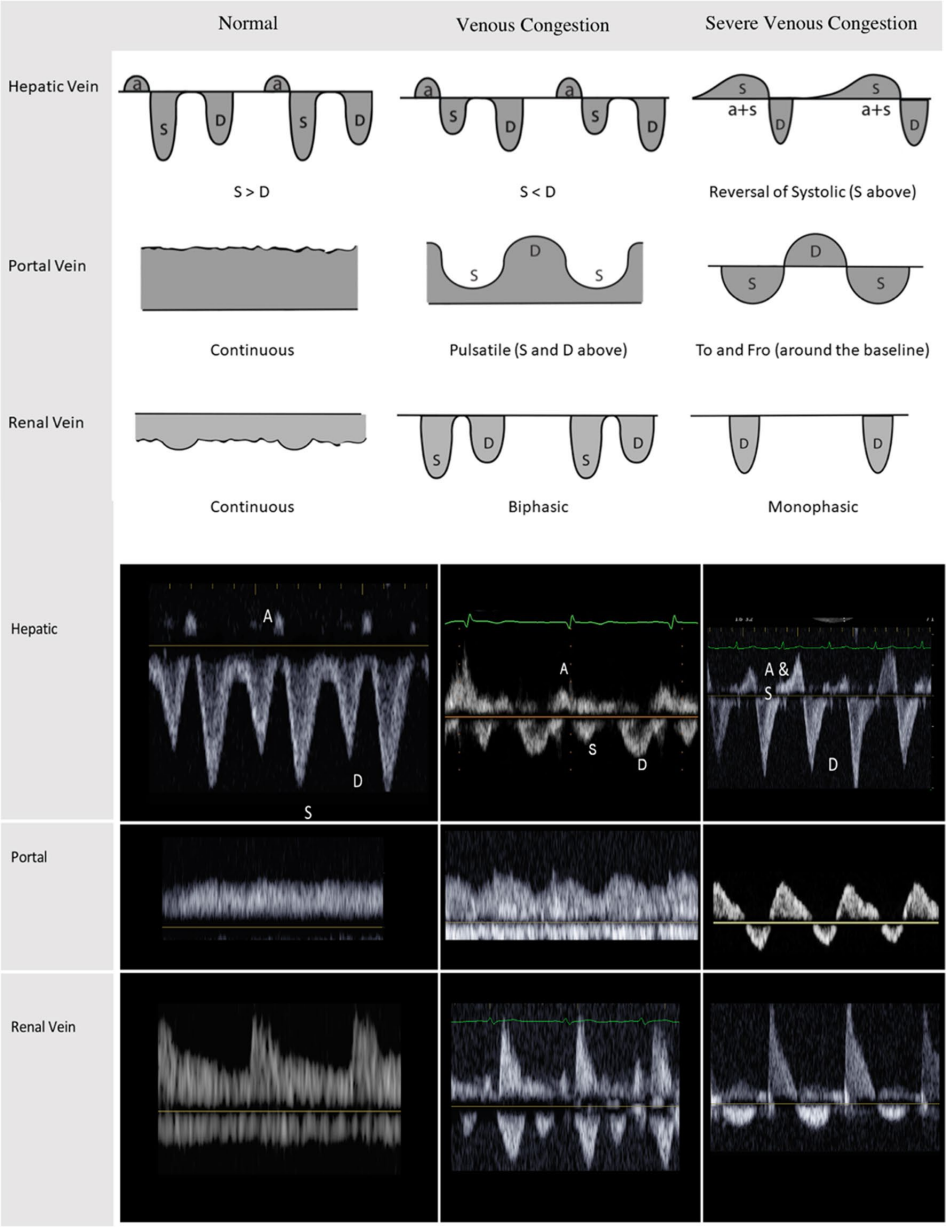
Normal and abnormal venous Doppler waveforms

The normal waveform is triphasic with three components, a retrograde A wave, a large antegrade S wave and an antegrade D wave (Fig. 2). The A wave occurs during atrial systole when right atrial pressure increases above the mean systemic filling pressure (MSFP), leading to retrograde flow away from the heart. Some describe a V wave as a correction back to baseline, but this is largely noncontributory. Once the right atrium relaxes and thus when the blood flow is normally the greatest, the S wave occurs during right ventricular contraction. The D wave occurs during right ventricular relaxation when tricuspid opening leads to a second drop in RAP. With normal RAP, the S is greater than D (S > D).

With an increase in RAP, the S wave decreases as RAP equilibrates with MSFP earlier. Due to this early equilibration, more RA filling occurs after the tricuspid valve opens leading to a relatively larger D wave. This is what is known as S-D reversal. As venous congestion worsens, the S wave becomes retrograde and often becomes continuous with the A wave leading to a biphasic pattern. Flow changes from normal to elevated; S > D, S < D, S above the baseline fused with A.

The HV was considered abnormal when the maximum negative velocity of the S wave was less than the maximum negative velocity of the D wave.

#### Portal vein Doppler

From a lateral costal or subcostal window, the portal vein is identified in the coronal plane using a phased-array transducer. Portal veins appear smaller and more hyperechoic than hepatic veins (Fig. 1b). CFD helps identify measurable blood flow. A PW Doppler gate is positioned in the middle of the vessel and the waveform is obtained, and the Doppler scale adjusted.

The normal waveform is a continuous monophasic flow above baseline with minor variations (Fig. 2). As venous congestion worsens, the portal flow becomes pulsatile, with decreases in flow during right ventricular systole. In extreme cases, flow can be interrupted or even have a retrograde component giving a to-and-fro appearance.

The PV were considered abnormal if the pulsatilty index was greater than 30%. The portal pulsatility index was defined as: (VMax − VMin/VMax) * 100%. Here, VMax is the maximal velocity and VMin is the minimal velocity during the cardiac cycle. We also examined PPI as a continuous variable to assess whether small changes in portal vein flow was a clinically important marker of venous congestion.

#### Intra-renal venous Doppler

From lateral costal window, the kidney is located in the coronal plane and a color flow box placed over the distal renal calyceal junction to cortex. (Fig. 1c) The PW Doppler gate is placed over interlobar vessels with a velocity set between 15 and 20 cm/s. The overall gain can also be used to try to see flow. It may be challenging to obtain in certain patients, and slow fanning of the probe may sometimes reveal better Doppler signals rather than relying solely on the color flow patterns. The waveform is usually considered adequate when both arterial (above the baseline) and venous (below the baseline) are seen clearly for two or more cardiac cycles.

Intra-renal venous Doppler is normally a continuous monophasic flow below the baseline (Fig. 2), which progressively becomes first interrupted with two phases, analogous to the S and the D waves of the hepatic vein flow. Similar to the hepatic vein pattern, as venous congestion worsens, the S wave becomes smaller and the D wave is more pronounced. Eventually the S wave disappears entirely, leaving only a monophasic D wave.

The intra-renal vein Doppler waveforms were considered abnormal if either a biphasic or monophasic renal vein flow pattern was present. This was defined as discontinuous venous flow with either a systolic/diastolic pattern or a diastolic only pattern.

All Doppler waveforms were reviewed by two members of the research team (RS and SM) blinded to clinical outcomes. Final venous waveform patterns were determined in this final review. Any disagreements were adjugated by a discussion between RS and SM. We calculated the inter-rater reliability between our reviewers. Patients in whom US images were missing or of insufficient quality to determine Doppler waveforms were excluded from the analysis concerning that specific waveform pattern.

### Outcome measurements and clinical data

Data were collected using manual and electronic data extraction methods from electronic health records. This included data on pre-enrollment renal function, demographic characteristics, diagnoses, SOFA scores, admission serum lactate values, intravenous fluids administered on the day of the FREE study, plasma electrolyte and creatinine values, need for receipt of renal-replacement therapy, CAM ICU score, fluid balance at admission and ICU discharge, need for mechanical ventilation and vital status at hospital discharge. Trial personnel performing data extraction did so using a standardized data extraction form and were unaware of group assignment.

The primary outcome measure is a major adverse kidney event (MAKE-30) [12]. The MAKE-30 is a composite outcome of an elevation of the creatinine level to ≥ 200% of baseline, need for renal replacement therapy or death. All outcomes were collected at the time of hospital discharge, death or within 30 days of admission to the ICU. Baseline creatinine values were determined using most recent creatinine values obtained during the year before hospitalization. If no baseline creatinine value was available and patients had no documented history of renal disease, the baseline creatinine level was estimated to be 1 mg/dL. We compared the rate of MAKE-30 events in patients with and without venous flow abnormalities in the hepatic, portal and intra-renal veins.

### Statistical analyses

#### Power analysis

The estimated rate of MAKE-30 in our cohort is 30%. From a previous internal review of patients undergoing FREE exams, the rate of MAKE-30 events in patients with venous waveform abnormalities is approximately 50%. Therefore, a cohort of 100 patients would identify a 20% absolute difference in MAKE-30 events between patients with and without venous waveform changes with an 80% power.

#### Regression analysis

Linear and logistic regression analyses were performed to assess the independent association of the HVD, PVD and RVD on the MAKE30. Variables were initially selected for logistic regression by comparing receiver operating characteristic curves (C-statistic) with the Delong test [13]. Secondly, stepwise selection of variables using criterion-based procedures was done to establish the best set of predictors in each regression model. The Bayes information criterion (BIC) was used to select the final model since this criterion usually results in more parsimonious models [14, 15]. For the logistic and linear regression models, multiple variables were tested. Multiple effect modification terms (e.g., age and SOFA) were created to detect statistical interaction.

All regression models were assessed with regression diagnostics. Logistic regression models were assessed for specification error using the “linktest” function in Stata, followed by goodness-of-fit testing using the Hosmer–Lemeshow goodness-of-fit statistic (computed as the Pearson chi-square from a contingency table of observed frequencies and expected frequencies), testing for multicollinearity and Pearson and deviance residual plot analyses to detect influential observations. Linear regression models were evaluated using the same tests for logistic regression. Additionally, outliers, leverage and influence were assessed using studentized residual plots, Cook’s D test, DFITS and DBETA, respectively. All tests were two-tailed, and a *P* value of < 0.05 was considered statistically significant.

Descriptive statistics were calculated to describe the cohort. Data are presented as means and standard deviations (SD) for normally distributed variables and medians and interquartile ranges (IQR) for non-normally distributed variables. Continuous measurements were compared with the student’s t test or Wilcoxon rank sum test as indicated.

## Results

From January 2019 to June 2019, we screened 167 patients for eligibility and 121 met inclusion criteria. Seven patients were excluded because of inability to obtain US images, or they had previously been enrolled in the study leaving 114 patients for the final analysis.

Males represented 59.7% of the cohort with a median age of 56.8 years. The most frequent ICU admission diagnosis was sepsis (19.5%). The majority of the patients were enrolled from the MICU (70.5%), followed by the SICU (14.7%), the trauma ICU (12.5%) and the neurotrauma ICU (2.7%). The median SOFA score on admission was 7; mean admission serum lactate was 2.8 mmol/L. Of the patients that required mechanical ventilation on admission (74.6%), 29.8% were on vasopressor or inotropic agents, and 21.9% demonstrated some degree of right ventricular disfunction (defined as a TAPSE < 1.6 cm) (Table 1). The median amount of IV fluids administered at the time of US evaluation was 636.42 mL (IQR − 415.59 to 2165.38 mL). The median fluid administered on the day of FREE evaluation in patients with and without MAKE 30 events did not differ statistically. Median fluid administered was 473.8 mL and 922.2 mL (*p* = 0.15), respectively.

**Table 1:** Demographics

Parameter	Mean or median^a^	SD or IQR or %
Age	56.8	16.7
Male (*n*, %)	68	59.7%
Race		
Caucasian	58	50.9
African American	49	42.9
Asian	2	1.8
Other/unknown	5	4.4
Comorbidities		
Heart failure	11	9.6%
Diabetes	30	26.3%
Chronic kidney disease	5	4.4%
Renal failure requiring dialysis	1	0.9%
AIDS	3	2.6%
Immunosuppression (not related to AIDS)	1	0.9%
*Admission vital signs*		
Systolic blood pressure	122.6	27.9
Diastolic blood pressure	69.1	16.2
Mean arterial pressure	88.7	19.6
Heart rate	95.9	21
Temperature (°F)	98.1^a^	96.8–98.6
Glasgow Coma Scale	10	7–15
*Admission physiological variables*		
SOFA score	7^a^	4–8
PaO_2_	142.8	77.9
Lactate	2.8	2.6
Right ventricular disfunction^b^	21.9%	95% CI 14.4–31.0%
*Critical care interventions (admission)*		
Mechanical ventilation	85	74.6%
Vasopressors/inotropes		
Norepinephrine	34	29.8%
Epinephrine	4	3.5%
Milrinone	1	0.9%
Phenylephrine	1	0.9%
Continuous renal replacement	14	12.3%

*SD* standard deviation, *IQR* interquartile range

^a^Median; ^b^defined as a TAPSE < 1.6 cm

On the FREE exam, the median SV was 52.3 mL, CO was 4.5 L/min, RA pressure was 7.4 mmHg. The median TAPSE was 2.13, and IVC size was 1.96 cm. 5.7% of patients had a RA pressure greater than 15 mm Hg, and 41.1% of patients had a CO < 4L/min. 21.9% of patients had a TAPSE less than 1.6. 46.0% had an IVC > 2 cm in diameter.

A MAKE-30 event occurred in 43 patients, 37.4% (95 CI 28.5–46.9%) of the entire cohort. Mortality at 30-days was 17.5% (95 CI 11.1–25.8%). Need for renal replacement therapy within the first 30 days was 12.3% (95% CI 6.9%–197). An increase in creatinine greater than 200% from baseline occurred in 15.8% (95% CI 9.6–23.8%). There were 9 (7.8%) patients who received CRRT without experiencing an increase in creatinine greater than 200%.

Overall, 27.6% of the patients demonstrated S to D reversal on their hepatic Doppler images; portal pulsatility index was > 30% in 41.1% of patients, with 22.4% and 2.4% of patients demonstrating biphasic and monophasic renal vein signals, respectively. Of the entire cohort 9 (7.9%), 19 (16.7%) and 29 (25.4%) of patients had inadequate hepatic, portal and renal vein Doppler studies. Agreement of image interpretation occurred in 80.4% of Doppler waveform analyses. Cohen k demonstrated moderate agreement at 0.6.

In patients with S to D reversal in their hepatic vein flow, the proportion who experienced a MAKE-30 event was 58.6% compared to only 27.6% of the patients without S to D reversal. Likewise, in patients with an increased PPI the rate of MAKE-30 was 46.2% compared to 28.6%.

Using univariable regression, changes in both hepatic vein Doppler and portal vein Doppler demonstrated a statistically significant odds ratio for predicting MAKE-30 events. S to D reversal in hepatic vein flow had a odds ratio of 4.15 (95% CI 1.54–11.23%) for an increase in the likelihood of a MAKE-30 event. Likewise, PPI, when assessed as a continuous variable, demonstrated a odds ratio of 1.021 (95% CI 1.0–1.044). PPI, when examined as a dichotomous outcome of 30%, had a odds ratio of 2.28 (95% CI 0.09 0.87–5.96%) for an increase in the likelihood of a MAKE-30 event, although this was not statistically significant. Abnormalities in renal vein Doppler was not associated with an increase in MAKE-30 events in this cohort.

In our multivariable regression model, both hepatic vein S to D reversal and PPI as a continuous variable demonstrated statistically significant increases in the rate of MAKE-30 events. S to D reversal demonstrated an odds ratio of 4.03 (95% CI 1.45–11.22), and PPI demonstrated an odds ratio of 1.030 (95% CI 1.002–1.06). When PPI was examined as a dichotomous outcome, it was associated with an increased odds ratio of MAKE-30, but in this case the confidence interval crossed zero (OR 2.28 95% CI 0.869–5.96). Biphasic or monophasic patterns seen on intrarenal venous Doppler were not associated with an increase in the rate of MAKE-30 events in this cohort (Table 2).

**Table 2:** Associated risk of MAKE-30 events with hepatic, portal and renal vein Doppler abnormalities

Variable	Odds ratio	95% confidence interval	*P* value	Sensitivity (%)	Specificity (%)
*Hepatic vein*					
Hepatic S < D	4.03	1.52–10.7	0.005	50.0	86.6
*Portal vein*					
PPI (continuous)	1.03	1.002–1.06	0.034	47.1	86.9
PPI (> 30%)	2.28	0.87–5.96	0.09	50.0	86.9
*Renal vein*					
Biphasic	2.29	0.69–7.58	0.17		
Monophasic	2.50	0.80–78.6	0.60		

We performed a sensitivity analysis examining only the patients with no signs of right ventricular disfunction on FREE exam as well as excluding patients who did not require mechanical ventilation. These analysis did not affect the results of our model.

## Discussion

This study demonstrates that obtaining right-sided venous flow patterns is feasible and predicts important clinical outcome in critically ill patients. To our knowledge, this is the first prospective observational study in general ICU population assessing the utility of hepatic, portal and intra-renal vein Doppler. It is also the first to compare different venous Doppler metrics on major adverse kidney events at 30 days.

Multivariant regression models both found that hepatic and portal venous Doppler flow changes are associated with an increase in rate of MAKE-30 events. Hepatic venous Doppler remained statistically robust even in patients without RV dysfunction, suggesting these findings are not just a marker of right heart failure. We did not find that renal Doppler was predictive of MAKE-30 events. This may be due to the technical difficulty in obtaining adequate renal vein Doppler signals. In our cohort, 25.4% of the patients had inadequate renal Doppler scans, reducing the power to detect a difference in outcomes.

Previous studies found similar results, also indicating that abnormalities in portal and hepatic vein Doppler signals are associated with an increased risk of AKI after cardiac surgery. Beaubien-Souligny et al. [7] examined portal vein Doppler in post-cardiac surgery patients and found that abnormalities in portal vein flow were associated with an increased risk of AKI (odds ration [OR] 4.31, confidence interval [CI] 1.50–12.35, *p* 0.007). Similarly, Eljaiek et al. [8] found that in post-cardiac surgery patients, abnormalities in portal venous flow were associated with an increased risk of AKI. Beaubien-Souligny et al [9] also reported that abnormalities in portal vein and intra-renal vein flow were associated with an increased risk of AKI in post-cardiac surgery patients. In our study, PPI was not as predictive as in previous trials, OR of 4.31 vs. 2.28. This may be due to the increasing complexity of our cohort and the multifactorial nature of AKI in a general ICU population.

We used the MAKE30 composite outcome to represent the incidence of AKI as its components were readily available in our EHR, and its use has been recommended by the National Institute of Diabetes and Digestive and Kidney Diseases work group on clinical trials in acute kidney injury [12, 16]. The etiology of renal injury is multifactorial. This study supports that venous congestion is one element. Right-sided venous congestion caused by elevated right atrial pressure can be detected by predictable changes in Doppler venous flow patterns [10]. This data and that of others show that these changes are also associated with increased renal injury in a variety of ICU populations. It is possible that the increased incidence of AKI consistently observed with fluid positivity could be partly explained by elevations in right atrial pressure from fluid resuscitation causing venous congestion. Hepatic and portal Doppler changes may indicate the point at which ongoing fluid resuscitation will cause further increases in right atrial pressure and worsening venous congestion causing end organ injury. Further research is needed to understand how factors like mean airway pressure, positive end expiratory pressure, central venous pressure combines with or influence the association observed between Doppler venous flow and renal injury.

There are several limitations to this study. It is a single-center study, using a convenience sample of patients admitted to the hospital. This enrollment pattern may have introduced a selection bias. Also, all scans were done by trained ICU fellows with a clinical and research interests in ultrasound. Further studies are needed to determine the accuracy of obtaining and interpreting the images in broader population of providers. Finally, as an observation cohort study a causal inference cannot be made from the observed association between venous Doppler abnormalities and MAKE-30.

## Conclusions

This study indicates that portal and hepatic flows may be clinical useful tools to help identify patients at risk for renal injury. It is likely that they are indicative of elevated right atrial pressure causing venous congestion, perhaps identifying patients in whom fluid resuscitation should be more limited. Further research is needed to evaluate the feasibility and utility of using these metrics as part of a comprehensive resuscitation strategy.

## Data Availability

We have made available all pertinent data and material.
